# Online English Teaching System under the Background of Epidemic Situation Based on Intelligent Feature Recognition Technology

**DOI:** 10.1155/2022/6569279

**Published:** 2022-05-31

**Authors:** Luoyun Chen, Weiwei Wang

**Affiliations:** ^1^Department of Humanities, Chongqing Metropolitan College of Science and Technology, Chongqing 402167, China; ^2^Chengdu Jiuzhou Electronic Information System Co.Ltd, Chengdu, Sichuan 610000, China

## Abstract

In order to improve the effect of online English teaching in the context of the epidemic, this paper combines intelligent feature recognition technology to carry out an online English teaching system in the context of the epidemic and greatly reduces the number of variants by selecting mutation operators with excellent performance. Moreover, in this paper, the mutation adequacy and the number of mutation operators are regarded as two objective functions, and the selection problem of mutation operators is generated into a two-stage optimization problem, and the above problems are solved by a genetic algorithm. In addition, this paper sorts the mutation branches and preferentially covers the mutation branches of the mutants corresponding to the mutation operators with higher mutation scores. The experimental study shows that the online English teaching system based on intelligent feature recognition technology proposed in this paper meets the actual needs of online English teaching in the context of the epidemic.

## 1. Introduction

Affected by the epidemic, the Ministry of Education has successively issued relevant policies to guide colleges and universities to carry out online teaching in an orderly manner and strive to achieve “substantial equivalence” in order to support colleges and universities to do a good job of “suspending classes without stopping teaching and learning.” In order to ensure the effective transmission of information within a limited time, some teachers have “fragmented” the knowledge content. Considering that the time required to organize teaching interaction is significantly longer than the time required for offline classroom teaching to achieve the same effect, some teachers have replanned the learning objectives and teaching content that can be covered during an online live broadcast. At the same time, some teachers design driven tasks and give more authority to students for autonomous learning.

At present, the teaching concepts widely recognized by teachers include “teacher-centered,” “student-centered,” and “goal-oriented.” Among them, “teacher-centered” and “student-centered” are often referred to in opposition. This study will examine how online teaching has affected teachers' teaching guiding ideology and teaching philosophy, especially whether and how teachers' understanding of “student-centered” has changed. Researchers from teaching management departments and teaching development institutions should continue to emphasize the “substantial equivalence” of online teaching and offline teaching. The promotion of the online and offline hybrid teaching modes that may come in the future should carry out educational research and practice in advance.

For a long time, college teaching has been in the form of face-to-face, and it faces many obstacles and difficulties from offline to online. For teachers, it is not easy to update teaching concepts and habits through relearning, and it is difficult to master new online teaching skills overnight, and online teaching puts forward higher requirements for teacher-student interaction. In terms of students, online education is characterized by the separation of teachers and students, the separation of time and space, and the separation of teaching, which requires students to have high learning autonomy and self-management and restraint ability. In terms of social recognition, the key lies in the quality of talent training in online teaching, but this requires a long feedback cycle, and there is also a lack of relevant data and information in research and practice.

This paper combines intelligent feature recognition technology to carry out an online English teaching system under the background of the epidemic to improve the effect of online English teaching and promote the effect of English teaching reform.

## 2. Related Work

Teachers' teaching investment is an important factor affecting students' academic investment, academic achievement, and teaching quality [1]. Existing studies have shown that the relationship between teachers' teaching investment and learning performance is not a simple positive correlation [2], teachers' high investment may not necessarily lead to students' high learning performance, and the effect of teaching investment on learning performance will be affected by factors such as students' motivation [3]. In the context of online education, on the one hand, the impact of teachers' teaching investment on students' learning performance will be weakened due to the separation of teachers and students in time and space; more energy to promote the reintegration of teaching and learning. Therefore, exploring how online teachers can effectively invest in teaching in order to improve students' learning performance to a greater extent is an important topic that needs to be studied urgently to explore online teaching laws and teaching management [4].

“Teaching engagement” mainly comes from the concept of “work engagement,” and similar concepts also include “teacher engagement” and “teachers work engagement.” Work engagement is a multidimensional concept, which is an individual's overall and all-around investment in work, and a concept that connects individuals, organizations, and work performance [5]. Research believes that work engagement reflects an individual's voluntary allocation of personal resources within the scope of tasks required by occupational roles [6]. Work engagement is defined as “organization members matching themselves with job roles through self-management,” which is reflected in the individual's energy and cognitive and emotional input in work [7]. Work engagement is defined as “a positive, well-rounded, work-related emotional and cognitive state characterized by vigor, dedication, and absorption.” The definition of energy, dedication, and focus corresponds to Kahn's definition of energy or behavior, emotion, and cognition, respectively, Saffili notes. This view of defining work engagement from behavioral, affective, and cognitive dimensions is widely accepted and has strong operability [8]. The teaching engagement is defined as the sum of emotion, time, and energy invested by teachers in teaching. “Teacher work engagement” and “Teacher engagement” is a concept with richer connotations than teaching engagement, which describes the work engagement of teachers in their occupational scope, such as the investment in teaching, teaching research, and professional development. [9]. Teaching input is the sum of the energy, emotion, and cognition invested by teachers in teaching work, which reflects the voluntary allocation of personal energy, emotion, and cognition of teachers within the scope of work tasks corresponding to teaching functions, with individual differences, dynamic balance, (fluctuates with changes in the teacher's internal and external environment), and plasticity (will be affected by other factors) characteristics [10].

Learning performance is an important variable to evaluate and test the quality and effectiveness of education and teaching, and it is also the core factor of distance education quality [10]. Learning performance includes completing objective learning objectives and learning achievement, satisfaction, and other subjective experiences related to learners. According to the existing definition, learning performance includes not only students' academic performance, but also their performance and emotional experience in learning activities [11]. According to the research needs and the convenience of data collection, existing research often uses academic performance and learning satisfaction as the measurement indicators of learning performance [12]. With the rise of situational learning analysis in online education, more and more studies have included learning engagement, which reflects the performance of the learning process, into the category of learning performance evaluation [13]. Learning engagement is an important variable affecting academic achievement [14]. Learning engagement is a multidimensional and complex concept. The more recognized structure of learning engagement includes behavioral, cognitive, and emotional engagement dimensions [15].

## 3. Intelligent Speech Feature Recognition Technology

The method of weak mutation testing can effectively reduce the execution cost of mutation testing. According to the weak mutation test criterion, the mutation statement is transformed into a conditional statement. Then, before instrumenting the original statement, the test data covering the true branch of the conditional statement must kill the corresponding variant. Therefore, when performing mutation testing, it is only necessary to cover the true branch of the conditional statement, which improves the efficiency of weak mutation testing.

We assume that the original program is *P*, the statement that implements the mutation operation is *s*, and after the mutation operator is implemented on the statement *s*, the obtained variant is *s*′, and a variant *m* is obtained by replacing *s* in *P* with *s*′. If a test data *t* can be executed to *s*′ in *m* and the state after executing the statement *s*′ is different from the state of *s*, then, according to the weak mutation test criterion, the variant *m* is killed. If the conditional statement *b*: if (*s*!=*s*′) is installed before the mutation statement of the original program, the test data of the true branch of the conditional statement *b* can be covered and the mutation *M* can be killed by the weak mutation test criterion. According to this principle, the variant killing problem in the mutation test can be transformed into a true branch problem covering the conditional statement *b* in the test.

In the test, as many conditional statements *b* can be constructed as there are variants, and the true branch of the conditional statement corresponds to the variant *m* one-to-one, and *b* is called the variant branch. The set of all mutation operators is *E*, and the selected set of mutation operators is denoted as *E*′. All mutation operators are implemented on *P*, and the resulting mutant set is recorded as *M*, *M*={*m*_1_, *m*_2_,…, *m*_*i*_,…*m*_|*M*|_}. Correspondingly, the constructed variant branch set is denoted as *B*, and *B*={*b*_1_, *b*_2_,…, *b*_*i*_,…*b*_|*B*|_}. It can be seen that |*M*|=|*B*|, and the true branch of *b*_*i*_ corresponds to *m*_*i*_, one-to-one.

We assume that the program under test is *P*, the set of operators is *E*, and it is set to *E*={*X*_1_, *X*_2_,…, *X*_*n*_}. At the same time, we assume that the mutation score of the variant corresponding to the mutation operator subset *E* before reduction is 100%, then the current problem can be described as a subset *E* of *E* with the fewest elements is found, so that the mutation score of the corresponding variant of *E* will not be significantly reduced.

It can be seen from the problem description that the purpose of selecting mutation operators is to make the number of mutation operators as small as possible when the mutation score is basically unchanged. Therefore, the number of elements contained in the mutation operator set *E′* is taken as the objective function, that is [16],

(1) $$\begin{array}{c} f\left(E^{\prime }\right)=\left|E^{\prime }\right|. \end{array}$$

The study found that using ABS, AOR, LCR, ROR, and UOI, these five groups of mutation operators can achieve a higher degree of mutation adequacy. These five types of mutation operators contain 58 specific mutation operators.

After selecting the mutation operator set *E*, if the random test data set can kill each variant in the variant set, then the mutation score corresponding to *E* can be expressed as follows:

(2) $$\begin{array}{c} MS\left(E^{\prime }\right)=\frac{k}{\left|M\right|}. \end{array}$$

The mutation score corresponding to the reduced mutation operator should not decrease. That is to say, the corresponding variant of the mutation operator after reduction and the corresponding variant of the original mutation operator can obtain the same mutation score, then the first constraint condition for the reduced mutation operator subset can be expressed as follows:

(3) $$\begin{array}{c} MS\left(E^{\prime }\right)=MS\left(E\right). \end{array}$$

The ease with which a variant is killed can be measured by the ratio of the number of test data to kill the variant to the number of test data that can be executed to the location of the variant, that is,

(4) $$\begin{array}{c} k_{m}=\frac{t_{k}}{t_{r}}. \end{array}$$

Here, *m* represents the mutant, *k*_*m*_ represents the killing rate of the mutant *m*, *t*_*k*_ represents the number of test data that kills the mutant *m*, and *t*_*r*_ represents the number of test data that can be executed to the location of the mutant.

The closer the statements before and after the mutation operator is implemented, the harder the resulting mutant is to be killed, and the closer *k*_*m*_ is to 0. Moreover, the semantic correlation of the variation before and after the variant sentence is negatively correlated with the kill rate of the variant. Therefore, from the dynamic point of view, the semantic correlation of the variation before and after the variant can be expressed as follows:

(5) $$\begin{array}{c} \text{coor}=1-k_{m}\text{ among }k_{m}\in \left[0,1\right]. \end{array}$$

Based on the above discussion, the mathematical model of the mutation operator reduction problem given can be expressed as follows [17]:

(6) $$\begin{array}{c} \min\;f\left(E^{\prime }\right)=\left|E^{\prime }\right|,\\ \text{s.t.}\;\left\{\begin{array}{c} MS\left(E^{\prime }\right)=MS\left(E\right),\\ E^{\prime }\subseteq E. \end{array}\right. \end{array}$$

An individual is a subset of the original set of operators.

We assume that the candidate solution of the mathematical model of the mutation operator reduction problem is *E*′ and encode *E*′. The encoding method is as follows.

Since *E*′ is a subset of the original operator set, for each test data *X*, *X* ∈ *E* or *X* ∉ *E* in *E*={*X*_1_, *X*_2_,…, *X*_*n*_}, there are

(7) $$\begin{array}{c} \beta_{i}=\left\{\begin{array}{cc} 1, & {\text{X}}_{i}\in {\text{E}}^{\prime }\\ 0, & \text{other} \end{array}i=1,2,\ldots ,n\right.. \end{array}$$

Through the above formula, you can get a 0-1 string of length *n Q*, *β*_1_, *β*_2_,…, *β*_*n*_. If two individuals do not contain exactly the same mutation operator set, then their corresponding 0-1 strings are also not the same. On the contrary, different 0-1 strings correspond to different mutation operator subsets. That is to say, there is a one-to-one correspondence between the mutation operator set and the 0∼1 string. Furthermore, *β*_1_, *β*_2_,…, *β*_*n*_ can be used as codes for individual *E*′. Therefore, an individual *E*′ can be represented as a 0-1 string *β*_1_, *β*_2_, ⋯, *β*_*n*_ of length *n*.

From formulae (1) to (3), it can be known that the value range of the objective function *f*(*E*′) is 0∼*n*, and the value range of the variation score *MS*(*E*′) is 0∼1. In addition, the model requires that the smaller the value of *f*(*E*′), the better, and the larger the value of *MS*(*E*′), the more likely the constraints are satisfied. Therefore, they need to be transformed reasonably. For the objective function *f*(*E*′), we have the following equation [18]:

(8) $$\begin{array}{c} \varphi \left(E^{\prime }\right)=\frac{f\left(E^{\prime }\right)}{n}\\ =\frac{\left|E^{\prime }\right|}{n}. \end{array}$$

In this way, the value of *f*(*E*′) can be mapped to the range of 0∼1, the smaller the value of *f*(*E*′), the closer the value of *φ*(*E*′) is to 0.

For the constraint *MS*(*E*′)=*MS*(*E*), the closer *MS*(*E*′) is to *MS*(*E*), the closer it is to the constraint. Therefore, the difference between *MS*(*E*) and *MS*(*E*′) can be used as a penalty term, and we have the following equation:

(9) $$\begin{array}{c} \xi \left(E^{\prime }\right)=MS\left(E^{\prime }\right)-MS\left(E\right). \end{array}$$

Then, the value of *ζ*(*E*′) is in the range of 0 to 1.

According to the designed weight coefficient, the weighted combination of the objective function and the penalty function, the fitness function is solved, as shown in the following equation:

(10) $$\begin{array}{c} \text{fitness}\left(E^{\prime }\right)=\varphi \left(E^{\prime }\right)+\omega_{1}\xi \left(E^{\prime }\right). \end{array}$$

Here, *ω*_1_ represents the weight coefficient of the penalty term. It can be seen from the fitness value function that the fitness value of individual *E*′ determines the expected result of solving the target. The smaller the value, the closer the solution target is to the expected value; on the contrary, the more the solution target deviates from the expected value. The optimization problem described by the fitness function can be formulated as follows:

(11) $$\begin{array}{c} \min\;\text{ fitness}\left(E^{\prime }\right)\\ \text{s.t.}\;E^{\prime }\subseteq E. \end{array}$$

To use a genetic algorithm to solve the problem, it is necessary to give suitable genetic operators, including crossover operator, mutation operator, and selection operator:

(1) *The Crossover Operator*: it uses single-point crossover to realize the exchange between individuals in the set.
(2) *The Mutation Operation*: it uses single-point mutation to realize the mutation operation for an individual in the set.
(3) *The Selection Operation*: in this paper, an individual selection method is obtained based on the semantic analysis of the mutation operator, that is, the improved roulette method. We assume that there are *n* individuals *E*_1_′, *E*_2_′,…, *E*_*n*_′ in the population, and the sum of the fitness of all individuals is
  (12) $$\begin{array}{c} F=\sum_{i=1}^{n}\text{fitness}\left(E_{i}\right). \end{array}$$

The probability that the *k*-th individual chooses is

(13) $$\begin{array}{c} p_{k}=\mathrm{coor}\cdot \left(1-F\right)=\mathrm{coor}\cdot \frac{\mathrm{fitness}\left(E_{k}\right)}{F}. \end{array}$$

The closer the sentences before and after the mutation operator is implemented, the more difficult the resulting mutant is to be killed, and the closer the value of coor is to 1, the higher the probability of selecting the kth individual.

The cumulative probability of the kth individual is

(14) $$\begin{array}{c} q_{k}=\sum_{j=1}^{k}p_{i}. \end{array}$$

*q*_0_=0 is set, and a number is randomly selected from 0 to 1, which is recorded as *μ*_0_. If *q*_*i*−1_ < *μ*_0_ ≤ *q*_*i*_, the *i*-th individual is selected, and so on until the selected individual reaches the required number.

According to the above-given strategy, the main steps of using a genetic algorithm to solve the mutation operator reduction problem are as follows:

 
*Step 1*: the algorithm performs parameter assignment and initial population coding. The algorithm sets the population size, termination algebra, crossover and mutation probability, selection probability, etc. In the process of encoding an individual population, the individual population is a subset of the existing operator subset.
 
*Step 2*: the algorithm performs population initialization. After completing the generation of the initial population *Pop*_1_, the individuals in the population are randomly generated, and the population size is given in the parameter setting link as the first generation population *t* = 1.
 
*Step 3*: the algorithm calculates the individual fitness value. The fitness value of each individual of the *i*-th generation population *Pop*_*i*_ is calculated by the fitness value function. The lower the fitness value, the closer the individual is to the expected value, and the easier it is to be selected and inherited as the next generation of population individuals.
 
*Step 4*: the algorithm determines whether the termination condition is satisfied. The algorithm judges the results and observes whether the population has a better individual or reaches the maximum evolutionary generation. If the population no longer produces better individuals, or the population has reached the maximum evolutionary algebra, the algorithm goes to step 6 to output the result. If the above-given conditions are not met, the algorithm goes to step 5 to continue the genetic algorithm procedure.
 
*Step 5*: the algorithm implements genetic operations. Crossover, mutation, and selection are carried out according to the genetic manipulation method to generate the *i*1 generation population. The algorithm transfers the new population to step 3 to continue the operation.
 
*Step 6*: the algorithm terminates. The algorithm selects the optimal result and outputs it.

After implementing all mutation operators on P, the resulting mutant set is denoted as *M*, *M*={*m*_1_, *m*_2_,…, *m*_*i*_,…*m*_*M*Δ_}; correspondingly, the constructed mutant branch set is denoted as *B*, *B*={*b*_1_, *b*_2_,…, *b*_*i*_,…*b*_*B*_}, and |*M*|=|*B*|. The test data set that needs to be generated is denoted as *T*. According to the idea of weak mutation transformation, five types of mutation operators in MuClipse are used to generate mutants. Then, it finds the equivalent variant by manual analysis, generates a variant statement with the help of mutation testing software, constructs a branch coverage statement, and loads the statement into the source program to obtain a new test program *P*′. Branch coverage statements can be obtained from the log file “mutation_log.”

For the tested program P, the constructed variant branch set is denoted as *B*, *B*={*b*_1_, *b*_2_,…, *b*_*i*_,…*b*_|*B*|_}, and the variant branch set sorted according to the above-given method is *B*′, *B*′={*b*_1_′, *b*_2_′,…, *b*_*i*_′,…*b*_|*B*|_′}. None of these mutated branches are covered until test data is generated. We assume that the test data set to be generated is *T*, this time, *T*=∅, and the test data generation method that the mutation branch is covered is: first, we take the front most branch statement *b*_1_′ as the coverage target branch and generate random data according to the defined range of the input, and these random data are used as the input test data *t*. Then, the test program *P*′ is executed. If the test data *t* covers the branch statement *b*_1_′ or other branch statements in the test program, it deletes these branch statements in the mutation branch set B and adds the test data *t* to the test data set T. It continues until all mutation branches are covered or the designed algorithm reaches the set maximum value. When the mutation branch set is completely covered, the corresponding test data set *T* is what we are looking for, and *B*′ becomes an empty set at this time.

The specific algorithm steps for covering the mutation branch set are as follows:

 
*Step 1*: according to the results of selecting mutation operators, the algorithm preferentially selects the variants corresponding to the operators with high mutation scores in the selection process and sorts the variants in descending order.
 
*Step 2*: the algorithm sorts the mutation branches corresponding to the above variants, and the sorted mutation branch set is *B*′, *B*′={*b*_1_′, *b*_2_′,…, *b*_*i*_′,…*b*_|*B*|_′}, so as to determine the order of covering mutation branches.
 
*Step 3*: the algorithm takes the mutation branch *b*_1_′ with the highest ranking order as the target branch and generates the coverage *b*_1_′ test data *t*.
 
*Step 4*: if the mutation branch *b*_*i*_′ is covered, *b*_*i*_′ is eliminated from the mutation branch set *B*′, and the test data *t* is added to the test data set *T*.
 
*Step 5*: the algorithm determines whether the termination condition is reached. If so, the algorithm outputs the test data set *T*; otherwise, the algorithm goes to step 3 to continue running.

For the new program *P*′ after the unloading branch statement, the set of mutation branches covered after the test data *t* is executed is *f*(*t*), then the corresponding test data coverage mutation branch process is: in the input domain *D* of the program *P*′, the test data set *T*={*t*_1_, *t*_2_,…, *t*_*i*_,…*t*_*n*_} of *P*′ is found so that for any mutation branch *f*(*t*_*i*_), there is a test data *t*_*i*_, which can cover the mutation branch *f*(*t*_*i*_). The set of mutation branches covered by the test dataset *T* is *F*(*T*), *F*(*T*)=*f*(*t*_1_) ∪ *f*(*t*_2_) ∪ ⋯∪*f*(*t*_*i*_) ⋯ ∪*f*(*t*_*n*_). Moreover, according to the idea of weak mutation testing, original program statement analysis, and mutation branch construction. Before loading it into the original statement, the test data generation process is transformed into the problem of covering the true branch of mutation, which is easier to implement in programming. Therefore, the transformed problem is formulated as the following minimization problem:

(15) $$\begin{array}{c} \min\;\left\{B-F\left(T\right)\right\}\\ \text{s.t.}\;T\in D_{1}\times D_{2}\times \cdots D_{i}\times \cdots \times D_{n}. \end{array}$$

Here, *n* is the number of test data, and *D* represents the input domain of the program *P*′. When the test dataset covers all mutation branches in B, *B* − *F*(*T*)=∅.

According to the weak mutation test criterion, the excellent mutation branch is preferentially covered, and the mathematical model of the test data generation problem is established. Considering that when solving the problem, the traditional genetic algorithm has a chance to fall into the local optimal solution, which leads to the phenomenon of population aging and hinders the solving efficiency of the problem. In this section, the regenerated genetic algorithm will be used to solve the above-given model to obtain more reasonable and effective test data. This paper uses the most commonly used branch coverage test data to generate the fitness function, the sum of layer proximity and branch distance, that is,

(16) $$\begin{array}{c} \text{fitness}\left(t\right)=\mathrm{Approach}\left(t\right)+\text{normalize}\left(\mathrm{dist}\left(t\right)\right). \end{array}$$

If there is a new population, but the fitness value does not increase or decrease, the solution process will enter the local optimal solution, which will affect the efficiency of the solution. In order to describe this situation, we assume that the aging factor *q* is used to measure the degree of aging in the evolution of the genetic algorithm, *n*_*i*,*j*_ represents the *i*-th individual in the *j*-th generation population, and *N*_popt_ represents the number of test data of the *t*-th generation, then the whole *t*-th generation of test data integers can be expressed as ∑_*j*=1_^*t*^∑_*i*=1_^*N*_popt_^*n*_*i*,*j*_. The aging factor *q* indicates that in the Δ*t* generation population, the number of newly added valid test data is higher than the total number of test data, as follows:

(17) $$\begin{array}{c} q=\frac{\text{ the increased num of }tc\left(\Delta t\right)}{\text{ the total num of }tc}\times 100\%. \end{array}$$

The specific calculation formula can be expressed as follows:

(18) $$\begin{array}{c} q=\frac{\sum_{j=1}^{t+\Delta t}\sum_{i=1}^{N_{poop\;}}n_{i,j}-\sum_{j=1}^{t}\sum_{i=1}^{N_{popj\;}}n_{i,j}}{\sum_{j=1}^{t}\sum_{i=1}^{N_{popt\;}}n_{i,j}}\times 100\%. \end{array}$$

**Figure 1:**
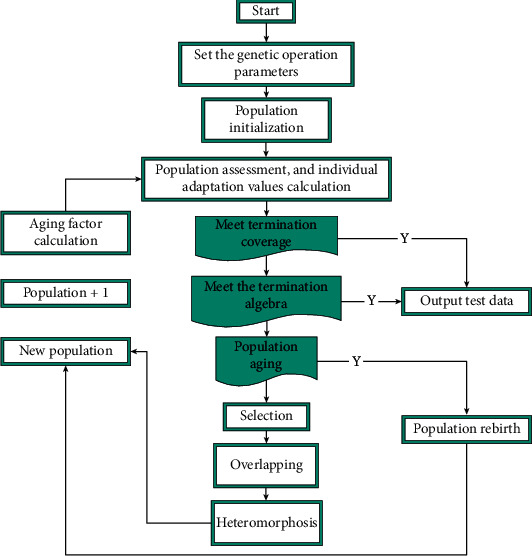
Flowchart of rebirth genetic algorithm.

For the branch coverage criterion, when the value *q*, which measures the aging degree of the population, reaches a threshold, the population cannot continue to evolve to increase the coverage fitness value, and the population is regenerated at this time. That is, the current population is discarded, and a new population is generated to continue the evolution and solution, which makes the genetic algorithm has a stronger ability to jump out of the local optimal solution. The flowchart of the Reborn Genetic Algorithm (RGA) is shown in Figure 1.

In this paper, based on the genetic algorithm of regeneration, aiming at the specific problems of test data generation, the specific steps are designed as follows:

 
*Step 1*: the priority strategy is covered by the mutation branch, the algorithm gives the descending order of the mutation branch of the tested program and sets the parameter values required by the regenerated genetic algorithm.
 
*Step 2*: the algorithm initializes the population and randomly generates test data in the input domain.
 
*Step 3*: the algorithm takes the population individuals as input, takes the branch statement with the highest ranking as the target branch, and executes the test program.
 
*Step 4*: the algorithm performs individual evaluation. If the individual of the population can cover the target mutation branch in the mutation branch set B, the covered branch is deleted and the individual is retained.
 
*Step 5*: the algorithm determines whether the termination condition is satisfied. If satisfied, the algorithm goes to step 7 to output the result; otherwise, it continues to run.
 
*Step 6*: the algorithm pair determines whether the population is aging. If the population ages, perform a respawn operation. Otherwise, the algorithm performs crossover, mutation, and selection according to the genetic operation method to generate the next generation population. The algorithm transfers the new population to step 3 to continue the operation.
 
*Step 7*: the algorithm terminates. The algorithm selects the optimal result and outputs it.

## 4. Online English Teaching System under the Background of Epidemic Situation Based on Intelligent Feature Recognition Technology

**Figure 2:**
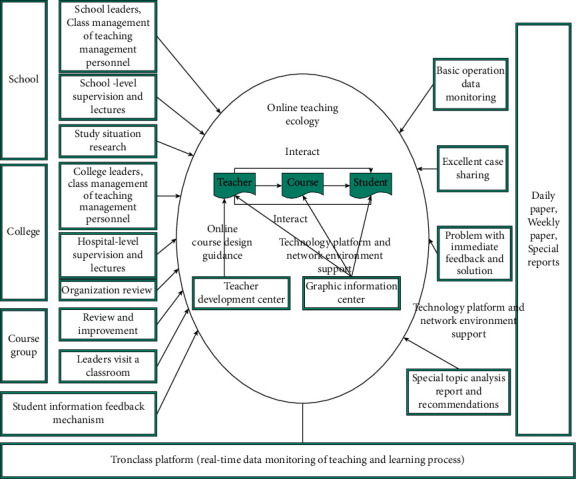
Online course teaching quality management system.

The school builds an online course teaching quality management system from different stages before, during, and after the course starts. From multiple perspectives such as students, teachers, and supervisors, data analysis and information disclosure are carried out on the operation of the teaching process, background data, teacher-student feedback, etc., to continuously improve the quality of online course teaching (Figure 2).

**Figure 3:**
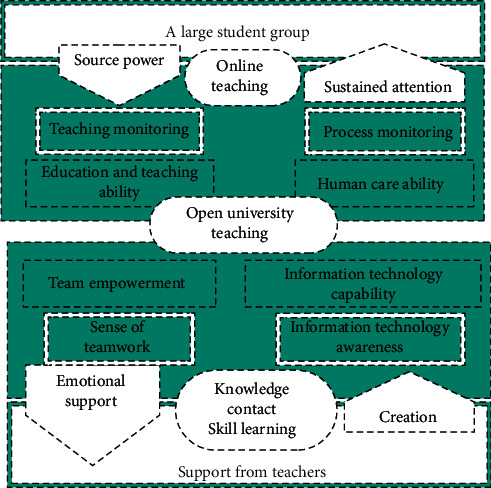
Teachers' online teaching ability model.

From the perspective of practical knowledge, the online teaching ability model of teachers is built on the basis of the relationship between categories (see Figure 3). The generation path of practical knowledge is the soil for model construction. In the practice of education and teaching reform, practical knowledge is generated in the path of teachers' action and reflection through certain problem situations. The externalized teacher's practical behavior is internalized into the teacher's ability, and then the tacit knowledge is externalized through qualitative data collection methods such as interviews to provide support for the construction of the ability model. The research on teachers' practical knowledge is from the observable and measurable external behavior of teachers to the structure and reasoning of deep teachers' inner cognition and then to the ethical dimension that constitutes the internal force of practical knowledge.

**Figure 4:**
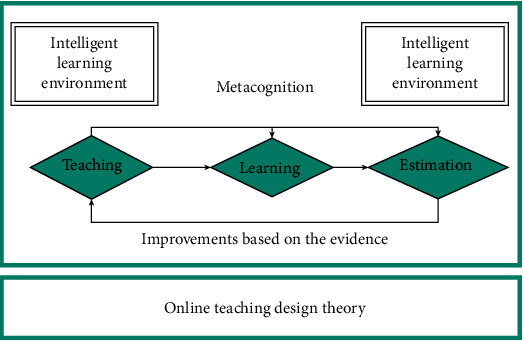
“Learning-teaching-evaluation” integrated online teaching application system.

This paper proposes an online teaching application system integrating “learning-teaching- evaluation” in primary and secondary schools (as shown in Figure 4).

**Figure 5:**
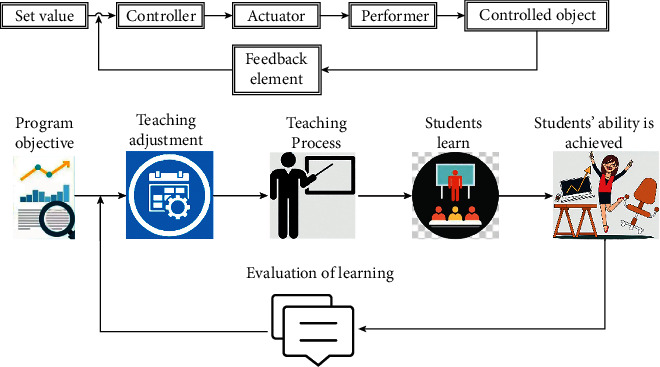
Analogy between teaching process and typical control structure.

The teaching concept based on OBE can be in a one-to-one correspondence with the classical feedback control theory. The controlled object of teaching is equivalent to the learning effect of the students, the set value is equivalent to the course goal, the signal processing unit is equivalent to the learning state evaluation system, and the controller is equivalent to the dynamic adjustment of the teaching process, and the feedback loop is equivalent to the student learning status feedback. The whole process is shown in Figure 5.

**Figure 6:**
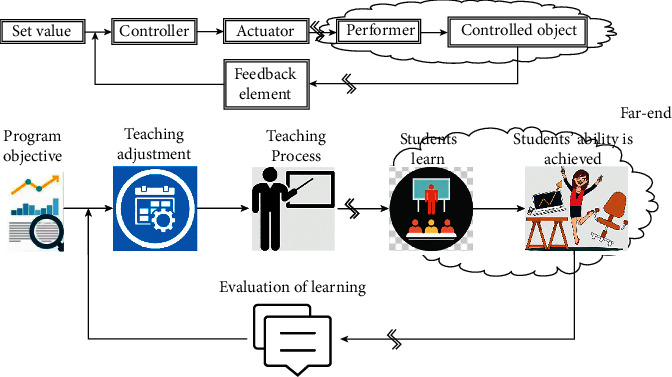
Schematic diagram of the closed-loop achievement of course objectives in the online teaching mode.

For teachers, it is equivalent to putting students' learning process and students' ability at the remote end, and the execution process and learning effect of learning have to be delayed due to the physical isolation of the network. Teachers' teaching process cannot be adjusted in real time according to students' learning status. The online teaching mode has the influence of network delay and uncertainty factors on both the forward and feedback paths, as shown in Figure 6.

**Figure 7:**
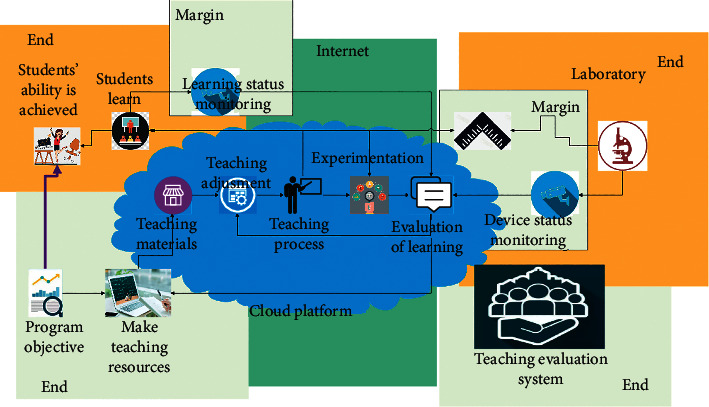
Deployment model of three-terminal collaborative student and equipment closed-loop OBE online teaching platform based on cloud control.

Figure 7 shows the closed-loop online teaching mode for students based on cloud control. Its goal is to solve the following online teaching problems to achieve substantially equivalent teaching effects. This paper uses the student-side camera to detect the student's state in real time, applies artificial intelligence, pattern recognition, and other technical methods, and integrates multiple heterogeneous data such as student's posture, learning time, and the noise of the learning environment to evaluate and predict the student's learning state. Moreover, this paper dynamically adjusts the teaching process according to the evaluation results of students' learning status. For the online teaching method, it can display the percentage of students who are excited, fatigued, and distracted, thereby prompting teachers to adjust the course progress and rest time. For the teaching method of course video recording, state prompts are given to students, or the video pause time is dynamically adjusted. According to the evaluation of students' learning status, a comprehensive score of classroom performance is given and included in the total score of students, which is substantially equivalent to the classroom performance score of offline teaching.

**Figure 8:**
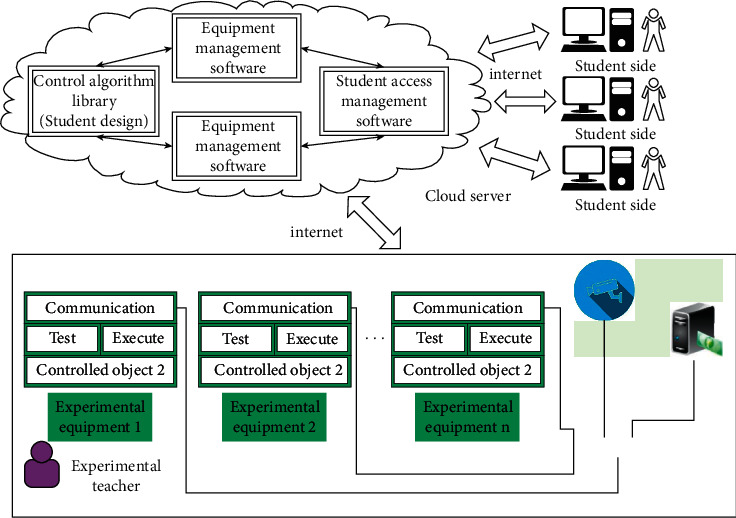
Online experimental platform for equipment based on cloud control.

The cloud controller architecture is deployed in the cloud and the network delay and uncertainty compensation are added to simulate the control effect of the local controller. With the video guidance of the instructor on the laboratory site, most of the experiments related to the core control theory and control algorithms of automation, electrical engineering, and automation can be completed. The realization structure of the online teaching experiment platform based on cloud control is shown in Figure 8.

**Figure 9:**
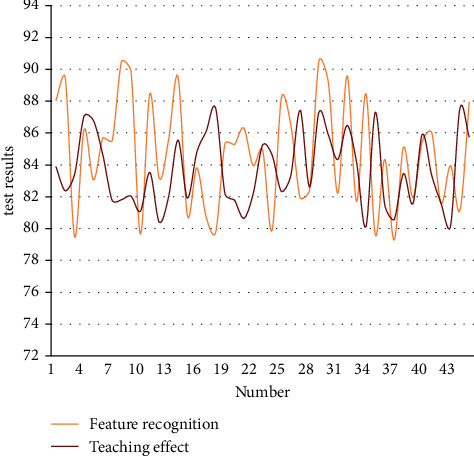
Statistical diagram of the effect of the online English teaching system based on intelligent feature recognition technology.

The above constructs an online English teaching system based on intelligent feature recognition technology. On this basis, the effect of the online English teaching system based on intelligent feature recognition technology proposed in this paper is verified, and the feature recognition effect and English teaching effect are verified, respectively, and the results are shown in Table 1, and Figure 9 are obtained.

The above-mentioned research shows that the online English teaching system based on intelligent feature recognition technology proposed in this paper meets the actual needs of online English teaching in the context of the epidemic.

## 5. Conclusion

The internal management system and organizational form of colleges and universities are a greater challenge for online teaching. Moreover, the bureaucratic organization and administrative orientation of colleges and universities do not completely match the flat, highly flexible, and flexible organizational environment required by online teaching. The above-mentioned difficulties make the “suspending classes and nonstop learning” of domestic colleges and universities under the new crown pneumonia epidemic is tantamount to an “encounter.” This article objectively describes the school's long-term training and preparation, prior deployment and arrangements, practices and evaluations in the process, and review and evaluation of gains and losses under this “encounter.” Moreover, this paper summarizes and reflects on the development process of online teaching transformation. In addition, this paper combines intelligent feature recognition technology to carry out an online English teaching system under the background of the epidemic, so as to improve the effect of online English teaching and promote the effect of English teaching reform. The experimental research results show that the online English teaching system based on intelligent feature recognition technology proposed in this paper meets the actual needs of online English teaching in the context of the epidemic.

## Figures and Tables

**Table 1:** Effect verification of online English teaching system based on intelligent feature recognition technology.

Number	Feature recognition	Teaching effect	Number	Feature recognition	Teaching effect	Number	Feature recognition	Teaching effect
1	88.03	83.89	16	83.81	84.79	31	82.23	84.34
2	89.46	82.38	17	80.71	86.11	32	89.58	86.47
3	79.48	83.42	18	79.75	87.56	33	81.70	84.16
4	86.22	87.06	19	85.34	82.18	34	88.47	80.11
5	83.06	86.78	20	85.28	81.79	35	79.61	87.29
6	85.69	84.62	21	86.31	80.64	36	84.34	81.50
7	85.55	81.77	22	83.95	82.18	37	79.28	80.56
8	90.53	81.82	23	84.93	85.24	38	85.11	83.45
9	89.85	82.03	24	79.88	84.64	39	81.95	81.57
10	79.66	81.10	25	88.19	82.34	40	85.48	85.91
11	88.46	83.52	26	86.52	83.32	41	86.02	83.35
12	83.17	80.41	27	81.93	87.42	42	81.60	81.57
13	85.59	82.00	28	82.43	82.62	43	83.95	80.14
14	89.52	85.55	29	90.52	87.30	44	81.14	87.53
15	80.82	81.92	30	89.17	85.88	45	87.95	85.74

## Data Availability

The labeled dataset used to support the findings of this study are available from the corresponding author upon request.
